# Evolution of Protein Structure and Stability in Global Warming

**DOI:** 10.3390/ijms21249662

**Published:** 2020-12-18

**Authors:** Sailen Barik

**Affiliations:** 3780 Pelham Drive, Mobile, AL 36619, USA; barikfamily@gmail.com

**Keywords:** global warming, secondary structure, thermophile, extremophile, hydrophobic interaction, protein structure, tryptophan, disulfide bond, mutation

## Abstract

This review focuses on the molecular signatures of protein structures in relation to evolution and survival in global warming. It is based on the premise that the power of evolutionary selection may lead to thermotolerant organisms that will repopulate the planet and continue life in general, but perhaps with different kinds of flora and fauna. Our focus is on molecular mechanisms, whereby known examples of thermoresistance and their physicochemical characteristics were noted. A comparison of interactions of diverse residues in proteins from thermophilic and mesophilic organisms, as well as reverse genetic studies, revealed a set of imprecise molecular signatures that pointed to major roles of hydrophobicity, solvent accessibility, disulfide bonds, hydrogen bonds, ionic and π-electron interactions, and an overall condensed packing of the higher-order structure, especially in the hydrophobic regions. Regardless of mutations, specialized protein chaperones may play a cardinal role. In evolutionary terms, thermoresistance to global warming will likely occur in stepwise mutational changes, conforming to the molecular signatures, such that each “intermediate” fits a temporary niche through punctuated equilibrium, while maintaining protein functionality. Finally, the population response of different species to global warming may vary substantially, and, as such, some may evolve while others will undergo catastrophic mass extinction.

## 1. Introduction

The rapidly accelerating pace of climate change is most pronounced as a rapid rise in global temperature and the serious harm it is inflicting on the environment and life, threatening our very existence on Earth [1,2,3,4,5,6]. The “global warming”, as the temperature change is commonly called, has drawn worldwide attention for decades and is rapidly approaching the “tipping point” [7,8,9]; unfortunately, however, multiple attempts to generate a universal consensus to combat it have also failed, mainly for short-term economic and political priorities [10,11,12]. Most experts now believe that global warming will soon become irreversible, or may already have, which means that it is, or soon will be, too late for preventive measures to reverse the process. In either case, it is safe to assume that global warming is here to stay, and, therefore, we must find ways to survive it. Considering this premise, it is important to understand how high temperatures affect living beings, and how they cope with it.

The majority of organisms, including all vertebrates, such as humans, grows in moderate ambient temperatures and are called “mesophilic” (less often, “neutrophilic”). The heat-tolerant organisms are classified as either “thermoresistant’ or ‘thermophilic”. The thermoresistant organisms require ambient temperatures for optimal growth, but can survive higher temperatures to some extent. The thermophilic organisms, in contrast, have an obligatory requirement of high temperatures for growth; however, they may retain viability at lower temperatures. Some investigators also use the terms “extreme thermophile” and/or “hyperthermophile” in describing those that grow at significantly higher temperatures, some approaching that of boiling water. In general, albeit somewhat arbitrary, the guideline range of optimal growth temperature is as follows: mesophile, 20–45 °C; simple thermophile, 50–64 °C; extreme thermophile, 65–79 °C; and hyperthermophile, ≥80 °C [13,14]. All high-temperature organisms belong to the larger family of “extremophiles”, defined as organisms residing in environmental conditions that are extreme in some respect, such as extreme cold (Arctic and Antarctic), alkaline or acidic pH (hot springs and mudpots), high salinity (oceans and various lakes) or pressure (deep sea), and desiccation (deserts). Reciprocally, the residents of extreme cold, the psychrophiles, as they are called, are defined as living below 15 °C; while they present an interesting contrast to the thermophiles in terms of both habitat and molecular mechanism, they are beyond the scope of this review. An organism can also be a crossover extremophile or “polyextremophile”, surviving and growing under multiple extreme conditions, such as cold and pressure, or heat and acidity [15]. In fact, oftentimes, the habitats of the extreme thermophiles combine high temperature with some of the other extreme conditions, both physical and chemical, aerobic or anaerobic [16]. The different classes of extremophiles may share some molecular mechanisms, such as those governing protein folding, but this review focusses specifically on high heat, which is most pertinent to global warming and its effect on terrestrial species, including humans. Lastly, unless otherwise mentioned, all types of thermoresistant and thermophilic organisms are addressed synonymously, or simply referred to as thermophiles, for the sake of brevity.

## 2. Rationale of Choice of Heat-Relevant Organisms and Macromolecules

### 2.1. Choice of Organisms

As mentioned above, unicellular protobacteria and archaea constitute a large fraction of thermophiles, but the hyperthermophilic organisms are mostly archaea [17]. Very few eukaryotes are thermophiles; however, some fungi grow at 50–55 °C [18]. Nevertheless, a significant amount of information is available on the heat shock response of eukaryotes. In fact, it is fair to assume that every organism is exposed to higher-than-ambient temperature at some point in its life, and, therefore, the response to heat is likely to be universal, and subsets of the response genes are phylogenetically conserved. Early evidence documented the survival benefit of heat shock to heat tolerance, i.e., when the heat shock response genes are induced by high but nonlethal temperature, the cells can survive a subsequent exposure to lethal temperature, which can be viewed as “stress immunization”. The technical advantage of the heat shock response is that it can be applied to mesophilic organisms that are easily grown in the laboratory and then shifted to desired higher temperature for defined periods, providing a detailed kinetics of gene induction and potentially, a glimpse of the regulatory circuitry that connects them. Natural thermophiles, on the other hand, are often polyextremophiles that must be grown not only at high temperature but also in specially reconstituted media and gas mixture. Ergo, the more extreme the natural habitat of the organism is, the harder is it to culture. The *Archaea* are particularly difficult to use due to their relatively exotic habitats and rudimentary genetics, and possibly unique genomic features (see later).

Heat denatures a protein by destroying the noncovalent bonds and altering the protein’s higher-order structure [19]. Evidently, an understanding of the effect of heat on a protein belongs to the realm of protein folding, which is a highly complex process and still not fully retractable—even less predictable—despite remarkable theoretical and technological achievements [19,20,21,22]. Although folding and refolding can be achieved with purified proteins in vitro, the role of the intracellular environment and interacting proteins, such as chaperons [23,24], which are relevant for a real-life survival, cannot be ignored. Many proteins also interact with RNA, both large and small, and a large variety of small molecules can act as regulators and modifiers [25,26]. Given these caveats, we should at least focus on studies comparing orthologous proteins in closely related species. For example, a major survival mechanism adopted by some bacteria (such as *Bacillus* sp.) is the formation of spores at high temperature, which occurs via a unique but well-researched gene expression program [27]. However, sporulation is a monopoly of select organisms and is of little use to others. In the higher eukaryotes, such as humans, live animal experiments are ruled out, and, therefore, results are obtained from in vitro studies of cell lines and proteins. In this review, relevant organisms and proteins are adhered to as much as possible, although the molecular principles of protein folding, which are fundamental to all life, were gathered from diverse organisms and summarized.

### 2.2. Choice of Proteins and Their Analysis

Early studies showed that enzymes, purified from thermophilic organisms, exhibit high temperature optima, comparable to the optimal growth temperature of the parent organism [28,29]. Moreover, a majority of the hyperthermophilic enzymes, recombinantly expressed in mesophilic hosts, retain all of their native properties, such as catalytic parameters [30,31,32] and three-dimensional structure [33,34,35,36]. These results reinforced the fundamental tenet that thermophilia, likely a function of the higher-order structure, is encoded in the primary structure (amino acid sequence) of a protein, and opened the door for analysis of thermoresistance at the protein level. In parallel, much of our knowledge of thermosurvival also derived from studies of heat shock response (often synonymously called “stress response”) [37,38,39], mentioned earlier, in which organisms and cultured cells, shifted to higher temperature, induce the expression of a large set of genes for survival. Evidently, these genes can play a critical role in protection from global warming, as well, and, therefore, they are explored in this review. Other areas include mutational analysis of thermostability of specific proteins acquired through natural selection, site-directed mutagenesis and reiteration, and comparison between orthologs of differing thermotolerance. When multiple orthologs are available, multiple sequence alignment points to specific amino acids that may have a role; these are then interrogated by mutational analysis to identify the residues required for thermophilia, since some differences may be due to evolutionary selection for other functions. As suggested previously [40,41], all mutations found in a thermoresistant protein (compared to its mesophilic ortholog) are unlikely to have been selected for thermoresistance, i.e., some may be unintended consequence of other selections [42,43]. Proteins with assayable function, such as enzymes, are useful in this regard, as they can allow distinction of functional residues from thermotolerant ones, and also ensure that functionality is not compromised to achieve thermal stability in reverse genetic mutational studies [41,42].

### 2.3. Ancestral Sequence Reconstruction

**Figure 1:**
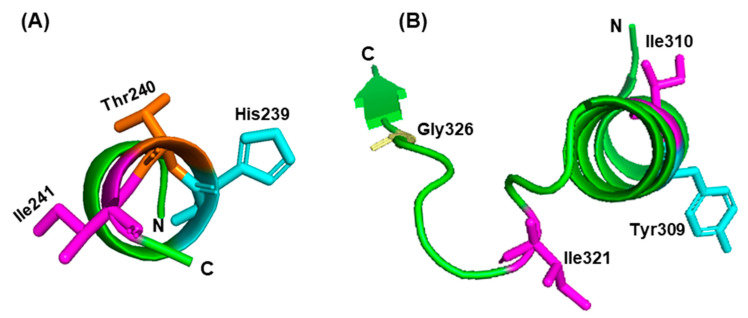
Two representative examples of ancestral sequence reconstruction (ASR), displayed in PyMol (https://pymol.org, Schrödinger, LLC, USA): (**A**) lignin peroxidase of *Phanerochaete chrysosporium*, a model white rot fungus [57], and (**B**) isocitrate dehydrogenase (ICDH) *Caldococcus noboribetus*, a thermophilic archaeon [58]. In both examples, only selected ASR-predicted mutations that led to a substantial increase in thermotolerance are shown, for brevity. The residues are colored, as well as named, and the peptide backbone is green. The model is based on the available crystal structures of the respective close homologs, viz lignin peroxidase (PDB 1B85) of *Phanerochaete chrysosporium* and ICDH of *Escherichia coli* (PDB 1CW1). Note that several but not all residues are located on helices. The directions of the amino (N) and carboxy (C) termini of the peptide backbone are indicated.

Even though global warming looms in the future, geobiological records from the past should facilitate our understanding of the mechanism and evolution of thermostability. A technical problem, however, is the scarcity of ancient biological material that is well-preserved, undegraded, and uncontaminated. Counter-intuitively, this is also true of cold-preserved material, such as the permafrost proxies in the Arctic that remain the least characterized [44]. An alternative, bioinformatic approach, named “ancestral sequence reconstruction” (ASR), has received significant attention [45,46,47,48,49]. It is based on the well-founded notion that the earth was extremely hot early in creation and gradually cooled down to allow the development of life. By inference, the earliest life forms must have been hyperthermophiles [50,51], which then evolved into simple thermophiles or mesophiles with the gradual cooling of the climate. This “hot-start” hypothesis [52] is supported by environmental evidence [53], as well as by the location of thermophilic organisms near the roots of the universal tree of life [54,55,56], such as the bacteria *Thermotoga*, *Aquifex*, and *Hydrogenbacter*, and many *Archaea*, such as *Thermoproteus* and *Thermococcus*. Thus, it is conceivable that extrapolation of an extant protein sequence, a few gigayears into the past, using a combination of molecular phylogenetic strategy, computer simulations, and maximum-likelihood methods, will lead to its ancient thermophilic or hyperthermophilic parent that is otherwise intractable, which is the essence of ASR [45,46,47]. The beauty of ASR is that it is testable, since the ancestral amino acids can be introduced into the extant proteins by recombinant means, and the resultant protein can be studied. In several such studies, an ASR-derived enzyme indeed exhibited higher thermal stability than the wild-type parent. In a representative example, the ASR-derived sequence of lignin peroxidase, a heme-containing fungal enzyme that has been extensively studied in *Phanerochaete chrysosporium*, was found to differ from the extant enzyme by eleven residues [57]. Eleven mutant enzymes containing each of these residues were then constructed and expressed in *Escherichia coli*; when the purified enzymes were assayed in vitro, several of them showed increased thermal stabilities and increased specific activities [57]. After 30 min incubation at 37 °C, the wild-type enzyme retained only 17% of its activity, whereas the single ASR mutants Ala110Gln, Gly198Ser, and Phe254 retained respectively 28.0, 33.5, and 18.4% of their activity. A triple mutant, in which three neighboring residues were all changed to ASR residues to generate the His239Phe/Thr240Leu/Ile241Leu enzyme, showed the highest thermoresistance, retaining 86.2% of its activity. The triple mutant also showed higher activity at its optimum temperature, i.e., specific activity of 68 units/mg at 40 °C, compared to the wild-type enzyme’s activity of 23 units/mg at 29 °C. When the authors examined the location of the residues on the crystal structure [57], residues 110, 254, and the 239/240/241 trio (Figure 1) were all found in helical regions, whereas only 198 was in a flexible loop. As we see later (Section 3), these locations agree with a role of helical stability in thermoresistance; they probably also improved the packing of the hydrophobic core of the peroxidase. The facts that these residues were far from the catalytic pocket, and that a few were located in non-helical regions, suggest that thermostability of a protein is dictated by the overall structure and not by any one residue.

In another study [58], the isocitrate dehydrogenase (ICDH) enzyme of *Caldococcus noboribetus*, a thermophilic archaeon that is often found in hot springs and hyperthermal vents, was subjected to ASR. Sequence comparison suggested a role of multiple mutations in thermostability, four of which were Tyr309Ile/Ile310Leu/Ile321Leu/Gly326Ser (Figure 1). These residues were located in multiple structures, including in a helix and in a flexible loop (Figure 1), documenting that diverse regions of a protein can contribute to thermostability. The half-inactivation temperature of the mutant was found to be ~93 °C, whereas that of the parent enzyme was 87 °C. Thus, use of ASR pointed to progenotes that were even more thermophilic than the *Archaea*, consistent with the high heat of the primordial environment. Similar studies with many other enzymes have further confirmed the conclusion that early organisms were indeed endowed with superior thermoresistance, adding confidence to ASR [50,59,60,61,62,63]. To the molecular biologist, the ASR studies provide an opportunity to understand the roles of specific amino acids in thermophilia; however, this requires analyses of additional proteins and determination of their structures. To the technologist, ASR offers a convenient and frugal route to design hyperthermophilic enzymes for industrial use (also see Section 4.2).

## 3. Role of Specific Amino Acid Residues and Interactions

As stated, the current review interrogates structural features and molecular interactions of proteins in the perspective of biological survival in global warming. Readers interested in the fundamental physicochemical principles of such interactions may consult several excellent reviews and articles published over the years [64,65,66,67,68,69,70].

### 3.1. Amino Acid Composition

A large number of studies have compared amino acid compositions of the proteome or a specific set of proteins in mesophiles and thermophiles [64,71,72,73]. The observed trend is that the thermophilic proteins favor charged, as well as large, hydrophobic residues, and also aromatic residues, but they disfavor uncharged polar ones. In a study spanning the temperature range from −10 to 110 °C, the amino acid set of Ile, Val, Tyr, Trp, Arg, Glu, and Leu (IVYWREL) was found to have a high correlation coefficient with optimal growth temperature [71]. The same study found a correlation of temperature with the total purine (A + G) content of the coding regions, somewhat matching with the IVYWRL composition.

The altered amino acid composition of thermophiles appears to be related to the altered overall nucleotide composition of the genomic DNA, which co-evolved with the translational machinery to prevent melting of the double helix at the higher temperature [14,71]. Higher proline content in proteins of bacterial thermophiles, for example, coincides with higher percentage of GC in the coding sequences, since CCN is the codon for Pro. Similarly, the hyperthermophilic archaeal genome is AG-rich, which matches with its codon bias, such as abundance of Thr (ACN codons) and Ser (AGU/C codons) [70]. The amino acids Asn, Gln, Met, and Cys are thermolabile for chemical reasons: While the amides (Asn and Gln) undergo deamidation in high heat, the sulfur-containing amino acids (Met and Cys) suffer from oxidation [74]. In thermophilic proteins, therefore, these amino acids are relatively sparse or internally buried, as we discuss later in the paper. Other studies showed that the archaeal thermophiles use an amino acid pattern slightly different from that of the eubacterial orthologs [75]; specifically, the archaeal thermostability was achieved by substituting non-charged polar amino acids (such as Gln) with Glu and Lys, and non-polar amino acids with Ile on the protein surface, which signals caution in comparing thermophilia across kingdoms, as mentioned earlier. Overall, codon bias and related translational components affect translational kinetics and in turn, the folding of the nascent proteins; this is a complex, multifactorial process, and thus, its correlation with thermophilia may not be straightforward [71,76].

Many of the thermophilia-determining residues are in critical locations in the secondary structure of the protein, several being located inside α-helices [71]. The thermophilic proteins tend to have a reduced α-helix content and deleted loops, while being richer in β-sheets [74,77,78]; however, this rule does not appear to apply to membrane proteins [71]. In general, unless specifically mentioned, the thermophilic signatures reported in published studies and discussed in most reviews [70] refer to soluble globular proteins. It is possible that incorporation of the helices of the membrane proteins into the lipid bilayer makes the mechanism of its thermostabilization different from that of the soluble ones [79,80]. Finally, for several amino acids, the hyperthermophilic signature differs from that of the simple thermophiles [70], which can only be attributed to the higher temperature (20–30 °C higher) to which the hyperthermophile cells are subjected to, requiring a higher molecular safeguard to prevent denaturation of the DNA helix and protein structures.

### 3.2. Role of Specific Amino Acids

The severity of the thermal effects on a protein hinges on the nature of the constituent amino acids. Several investigators have, therefore, searched for consensus amino acids in thermophilic proteins, and have indeed noted various signatures, in which certain classes of amino acids are either favored or disfavored for a variety of mechanisms [64,70,72,73,81,82,83,84,85,86,87,88,89,90], the predominant ones among which are summarized here. It should be mentioned that these observations are generalized, and that exceptions often exist, some of which have been noted.

(a) Charged amino acids (acidic and basic): The charged residues are hydrophilic, and in contrast to the hydrophobic ones, which are located to the center of the protein, are on the surface of the protein, where they promote interaction with the solvent and salt bridges with other moieties. The solvation and ionic interactions facilitate solubility and prevent heat denaturation and precipitation. In fact, an increase in surface ion pairs and a more strongly hydrophobic interior were concluded to be the two strongest contributors to thermostability [70,78,87,91,92,93,94].

(b) Bulky hydrophobic amino acids (Ile, Leu, Met, and Val): While the charged side chains are exposed to the exterior, it is the opposite with the hydrophobic ones, which are typically located to the interior of proteins through hydrophobic interaction, so that they are shielded from the solvent. This allows for tighter packing of the structure of a globular protein, making it more resistant to unfolding [95,96,97]. However, they are more prevalent in the hyperthermophiles than in the simple thermophiles, and their beneficial effect is more pronounced on larger proteins [98].

(c) Aromatic amino acids (Phe, Tyr, Trp, and His): The electron cloud above and below the plane of the cyclic structure sets the aromatic side chains apart from those of all other residues. They stabilize the core of the protein through interactions with other π electron clouds (another aromatic side chain within atomic distance) and with cations, which thwarts unfolding. However, to what extent this interaction contributes to thermophilia has been a matter of debate [70]. It appears that the simple abundance of aromatic amino acids is not a signature of thermophilia [99] and that the location is also critical; with Trp, for example, much of the observed difference relates to an increased number of Trp residues in α-helices and near helix termini (4.4% in mesophiles vs. 7.9% for thermophile protein orthologs) [84].

(d) Tryptophan (also see above): Although it is an “aromatic” amino acid, Trp is a unique amino acid in many respects [100], and it deserves special mention. It is the largest and rarest amino acid in the cell, has the largest π electron cloud in its side chain, and is produced by the most complex and energetically most expensive biosynthetic pathway. Besides Met (AUG), it is the only amino acid that is coded for by a single codon (UGG) in the standard codon repertoire. Thus, it stands to reason that Nature would use Trp sparingly, in only the strategically essential locations.

**Figure 2:**
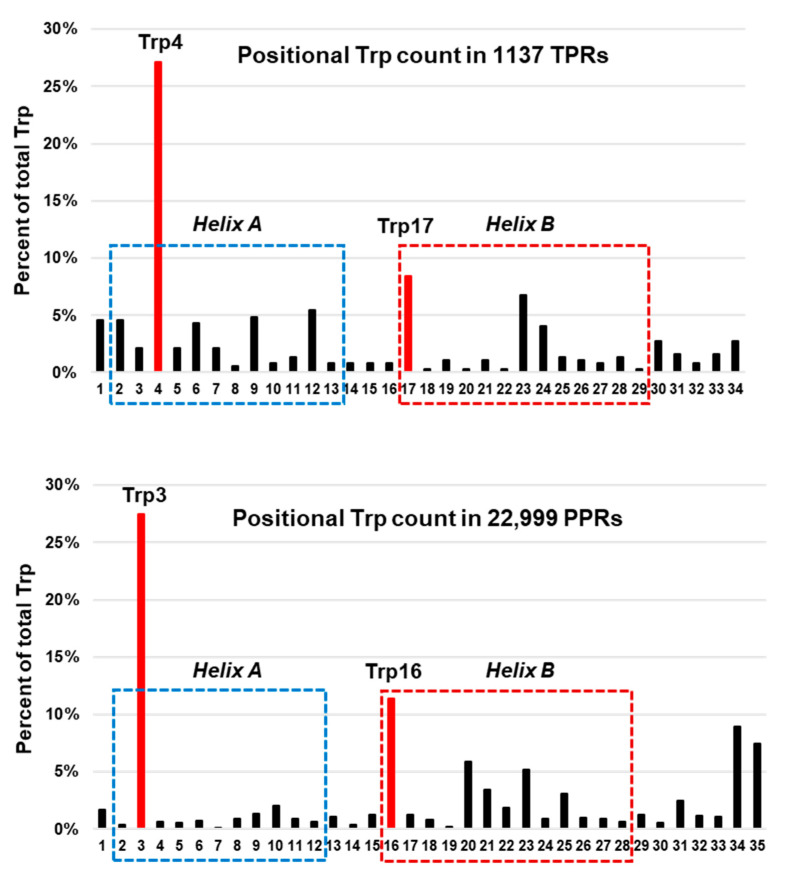
Near-terminal location of tryptophan (Trp) in all helical repeats. The published collections of the tetratricopeptide and pentatricopeptide repeat sequences [102,103] were visually analyzed for the location of Trp residues, and the numbers were plotted, using Excel. The two classic helices of the tetratricopeptide repeat (TPR) (**Top**) and pentatricopeptide repeat (PPR) (**Bottom**) motif are shown as Helix A and Helix B, and their amino acid position numbers are presented on the horizontal axis. The two most abundant Trp residues (Trp4/3 and Trp17/16, in Helix A and Helix B, respectively) are indicated as red bars; note that both are near the N-terminal end of the helices.

To test if the helix-terminal location of Trp is unique to thermophilic proteins, I analyzed the helices in a large number of helix-rich domains, namely the tetratricopeptide repeat (TPR) and the pentatricopeptide repeat (PPR) [101,102,103,104]. TPR and PPR are, respectively, 34- and 35-amino acid bi-helical motifs, found in diverse proteins that overwhelmingly belong to mesophiles [105,106,107]. Both repeats offer an excellent opportunity for this analysis because of the large number of helices they collectively offer. Moreover, they can be identified, and their secondary structure predicted with confidence, due to the conservation of signature amino acid residues [80]. A total of ~23,000 TPR and PPR sequences, representing >48,000 α-helices, were analyzed, and the largest concentration of Trp residues were found near the termini of the helices (Figure 2), confirming and extending early observations with different sets [102,107]. Thus, protection by helix-terminal locations are not unique to constitutive thermophiles, but can also help the mesophiles when temperature rises. It would be interesting to investigate the interacting partners of Trp, which may play critical roles in heat-stability.

**Figure 3:**
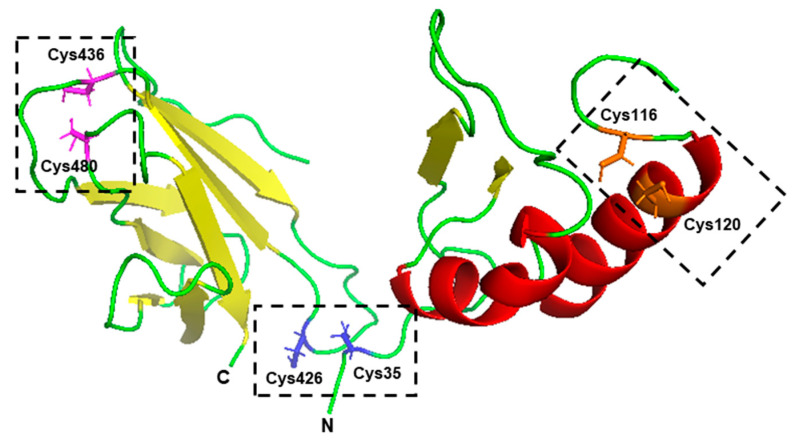
Site-directed mutagenesis to create Cys–Cys disulfide bonds that generated thermoresistance. The 3D structure of the triple-pair Cys mutant of *A. amylolytica* α-amylase was generated in silico, as described in Reference [116], and then subjected to homology-based I-TASSER modeling [117]. The modeling selected α-amylase (3BC9) of *Halothermothrix orenii* as a high “C-score” homology match among eight closest PDB templates (the other seven being 3BCF, 4JCL, 2DIJ, 2GJP, 6GXV, 4UZU, and 4JCL), independently confirming the previous studies [116]. The PDB structure, thus obtained, is displayed through PyMol (https://pymol.org, Schrödinger, LLC, USA). Each Cys pair, engaged in disulfide bonding and marked with a box, is shown as stick side chains and has a distinctive color: Cys35–Cys426 (blue), Cys116–Cys120 (orange), and Cys436–Cys480 (magenta). For the sake of clarity, portions of the structure and the disulfide bonds are not shown; moreover, no attempt was made to depict the proper rotamer of the side chains, and, therefore, the S atoms of a pair may not be directly facing each other in this representation. The N- and C-terminal directions are also indicated. The structural elements are color-coded: helix, red; strand, yellow; loop, green. A more detailed structure can be found in the original study [116].

(e) Acid amides (Asn and Gln): At physiological pH, deamidation of Asn and Gln is the most common nonenzymatic change in a protein that is promoted by elevated temperatures [98]. Deamidation increases the negative charge of the residue, which could cause structural change, unfolding and irreversible inactivation [108,109,110]. In fact, site-directed mutation of a strategic Asn to Asp has been shown to eliminate heat-mediated deamidation and inactivation of a fungal glucoamylase [111]. Asn and Gln are, therefore, highly disfavored in thermophiles, but more so in hyperthermophiles, since deamidation is more pronounced at higher temperatures.

(f) Aliphatic hydroxy-amino acids (Ser and Thr): A negative correlation between Ser/Thr content and optimum growth temperature has been noted [84], i.e., these amino acids are disfavored in thermophiles. Again, this is better correlated in hyperthermophiles than in simple thermophiles, suggesting that the negative effect is marginal when the temperature rise is small. It is also less pronounced in hyperthermophilic archaea [70].

(g) Proline: Pro is the only residue with an alicyclic secondary amine (-NH) in the side chain, to which the α carbon is a direct substituent. The NH loses the H in forming the peptide band, so that no hydrogen is available to donate a hydrogen bond, and, therefore, Pro disrupts an α-helix. On the other hand, the unique cyclic structure of the Pro side chain imparts conformational rigidity to the protein backbone, beneficial for thermotolerance. In other words, the introduction of Pro residues increases the thermal stability of a protein due to decrease in the entropy of the unfolded state [64,70,81,112]. It is, therefore, highly favored in hyperthermophilic organisms. The higher content of Pro, a helix-breaker, is in conformity with the results of an early survey of available 3D structures of thermophilic proteins in the PDB database, which revealed a significant increase in β-strands, compared to their mesophilic homologs [78].

(h) Disulfide and hydrogen bonds: The Cys–Cys disulfide bonds are covalent and hold a protein structure together, thus boosting thermal stability of proteins in which they occur [113]. For instance, the intramolecular disulfide linkages of the capsid protein of betanodavirus, a deadly pathogen of young marine fish, plays an important role, not only in the assembly of the virion, but also in its thermal stability [114]. However, not all Cys residues in a protein engage in disulfide bonds, since it requires spatial proximity of two Cys residues, and, therefore, occurrence of Cys alone does not predicate thermostability. Indeed, attempts to stabilize proteins by the introduction of extra Cys–Cys disulfide bonds through site-directed mutagenesis of recombinant proteins have yielded inconsistent results, improving the stability and temperature optimum (Tm) for some locations, but not in others [115,116]. In a representative study [116], when seven residue-pairs in the alkaline α-amylase from *Alkalimonas amylolytica* were replaced with cysteines, only three of them (C35–C426, C116–C120, and C436–C480) showed significant enhancement of thermostability (Figure 3), while the remaining four actually decreased it.

The Tm of C116–C120 amylase activity was ~5 °C higher than that of the wild type (50 to 55 °C), where some other pairs (not shown) had a lower Tm. A triple mutant, C35–C426/C116–C120/C436–C480, showed a six-fold increase in half-life at 60 °C and a 5.2 °C increase in Tm over the wild type. Note that the last pair, viz C436–C480, introduced alone, had little effect on the Tm.

These results clearly demonstrate that rational design of Cys disulfide bonds to improve thermotolerance is possible, but the effect is contextual and likely influenced by other intramolecular interactions; thus, the properties of the mutants need to be experimentally tested. Lastly, the disulfide effect should be most pronounced in oxidizing environments that prevent reduction of the disulfide bond.

In contrast to disulfide bonds, the hydrogen bonds (H-bonds) are weak, but they form between a greater variety of amino acid side chains, as well as with water, and thus provide significant strength to the overall structure. As a result, they are slightly more abundant in thermostable proteins; however, like disulfide bonds, their effect cannot be generalized [70,78,118]. Moreover, introduction of a new H-bond at one site may have a distant effect elsewhere in the structure, which is hard to predict.

**Figure 4:**
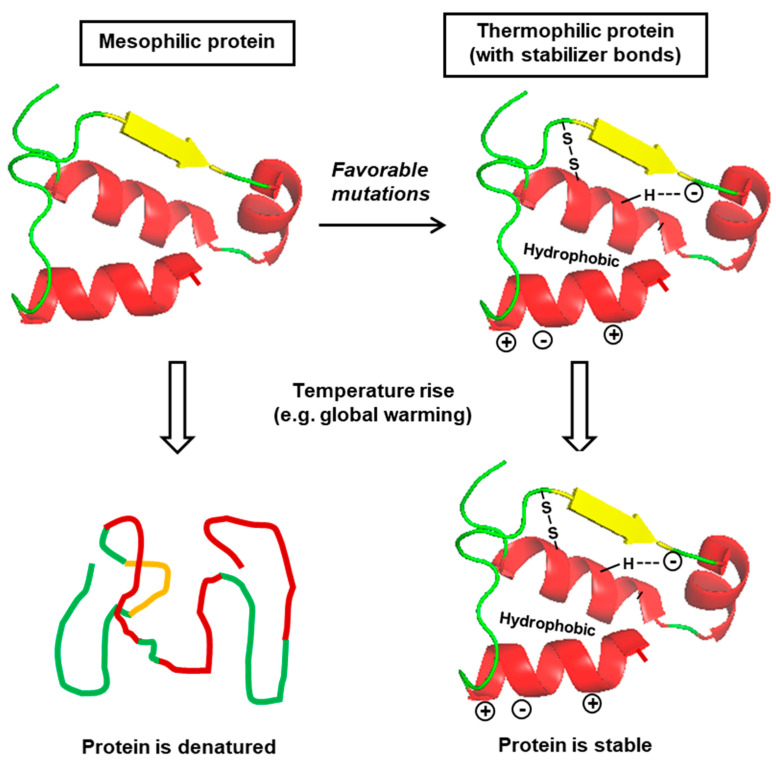
Diagram of the major consensus features for thermal stability in protein structure: disulfide and hydrogen bonds, charge–charge interactions on the exterior, and hydrophobic interactions in the interior (details in Section 3). These interactions stabilize the protein against inactivation/denaturation at high temperature. The secondary structural elements are color-coded, as in Figure 3.

In conclusion, there is no amino acid consensus that is universally and equally applicable to all thermotolerant proteins. Indeed, it is tempting to speculate that even the different thermotolerant mutant proteins, independently arising from the same mesophilic cell, will not contain the same mutations in the same genes [119]. Nevertheless, the broad and generalized features of thermotolerance are summarized here (Figure 4) and should serve as a useful reference.

## 4. Prospects of Mutational Survival in Global Warming

### 4.1. Designer Thermostable Organisms?

A futuristic and evolutionary question is whether we can harness the molecular knowledge of thermophilic mutations in designing proteins or creating organisms that will survive in global warming. In order to answer this query, we need to first determine how many mutations are needed to achieve useful thermotolerance. From the molecular differences described earlier, it is clear that stable and optimal thermophilia requires multiple mutational changes; however, there are a few examples where altering one or two amino acid(s) in a protein sometimes makes a difference, as described later. Another problem in designing a functional thermostable protein, particularly a large one, stems from the fact that the majority of missense mutations affects protein function [120]. This is because a single missense amino acid substitution can cause a free energy change of 0.5 to 5 kcal/mol, which most proteins can barely tolerate without losing stability [44,120,121,122]. The native functional state of a protein is thus marginally stabilized by the balance of the opposing forces of folding and unfolding [121,123]. Lastly, as alluded to earlier, structural rigidity improves thermal stability, but proteins also need to flexible in order to function and evolve. Thus, there is a trade-off between stability and flexibility, and directed evolution towards thermostability must balance between the two [124,125]. Designing of a thermostable organism from a mesophilic one is a quantum jump in complexity and requires many mutations of the proteome that also need to be mutually compatible; this may be fodder for science fiction but not a currently achievable goal.

On the other hand, spontaneous generation of a phenotype in simple and rapidly replicating unicellular organisms happens under all environmental challenges, and the same is true of temperature rise, as seen time and again in Nature and in laboratory conditions [126,127]. In an interesting early experiment [128], 3-isopropylmalate dehydrogenase (LeuB) of the extreme thermophile, *Thermus thermophilus*, was replaced with a temperature-sensitive chimeric LeuB. A thermostable mutant was then selected by culturing the transformant to higher temperature, and was found to contain Leu93 in the place of Ile93, which coincided with the Ile93 in the natural *T. thermophilus* LeuB. The mutant enzyme was confirmed to be more stable than the original chimeric enzyme. In another example [129], a mesophilic protein was converted to a highly thermostable form by changing the Glu3 and Glu66 to Arg and Leu, respectively. These were surface residues of the protein, which exemplify a simple and powerful approach for increasing the thermostability of a protein in small steps, on the way to higher levels of tolerance through multiple additional changes [70,130]. In a more realistic, tour-de-force experiment [131], mesophilic *E. coli*, grown in long-term culture, was subjected to increasing temperature, and the sequentially collected survivors were subjected to whole-genome sequencing. A correlative analysis of genotype and growth phenotype of the mutants identified the glpF and fab genes as being mutated in the high-temperature isolates. The glpF gene product is involved in glycerol transport and is essential for glycerol utilization; fab codes for a fatty acid desaturase/isomerase that adds double bonds to fatty acid moieties. While the glpF mutation was a stop codon at position three and, therefore, essentially did not make any protein, the glpF mutant had a single missense mutation that significantly lowered its activity. Thus, two metabolically important enzymes played the most critical role in organismic thermal resistance. These results provide strong indication that mesophilic organisms will be successful in producing temperature-resistant mutants under challenge. However, these studies also provided direct evidence that only a small range of temperature rise is tolerated, above which optimal metabolism and life cycle cannot be sustained. In other words, only a few degrees separate life from death, and this is why a rapid rise in global temperature may lead to extinction of many species, a process that has already begun for many nonhuman species, accompanied by a cascade effect [132]. Genetic changes in evolution take a generation to be inherited, which for larger animals with long life spans and slow reproductive maturity can be many years, making them particularly vulnerable.

### 4.2. Practical Applications of the Molecular Knowledge of Thermophilia

Although spontaneous natural mutations followed by survival of the thermotolerant ones will perpetuate some form of life on the planet, our molecular understanding of thermophilia and thermoresistance, boosted by the attention to global warming, has become profoundly useful in a multitude of industrial applications, such as high-temperature environmental cleaning, biodegradation, and process engineering. In recent years, thermophilic organisms and enzymes have been optimized for hydrolysis of various biopolymers [16,17,18,116,133,134]. The initial hurdle of the paucity of molecular genetic tools is being overcome [135], and the expanding list of thermophiles used for recombinant gene manipulation now includes, but is not limited to, several genera of bacteria (*Caldicellulosiruptor* and *Thermotoga*) and archaea (*Sulfolobus*, *Thermococcus*, and *Pyrococcus*).

Long-chain polysaccharides such as cellulose, starch, and pectin, do not enter cells, and, therefore, their utilization must precede hydrolysis to smaller units by extracellular hydrolases. Use of the cognate enzymes at high temperature allows access to bonds buried in the polysaccharide branches and tertiary structures. The enzymatic processing of starch into various sugars constitutes an important area in food, pharmaceutical, paper pulp, and textile industries. In the example shown earlier (Figure 3), the thermostability of alkaline α-amylases was successfully improved through rational in silico design to add Cys–Cys disulfide bridges in the catalytic domain [116]. Reciprocally, the naturally thermophilic chloramphenicol acetyltransferase enzyme from *Clostridium thermocellum*, a thermophilic anaerobic bacterium, was engineered to enable it to convert isobutanol into isobutyl acetate through site-directed mutation of the conserved Phe97 in the substrate-binding pocket to Trp, which allowed the mutant bacteria to produce isobutyl acetate directly from cellulose [133]. Structure-based mutagenesis like these can be applied to other enzymes, as well to improve either thermotolerance or catalytic parameters. In a different example, thermophiles are being engineered to degrade plastics, a perilous pollutant of the environment. Polyethylene terephthalate (PET), a major component of solid plastic waste, including microplastics, which cannot be easily degraded, partly due to its solid state [136]. However, effective enzymatic hydrolysis of PET can occur at a glass transition temperature >70 °C. In one study [134], *C. thermocellum* was genetically engineered to express extracellular thermophilic cutinase (LCC), isolated from a plant compost metagenome, which could degrade PET at up to 70 °C. In a pilot test, the resultant bacteria degraded ~60% commercial PET in 14 h of incubation at 60 °C (the optimum growth temperature of *C. thermocellum*), which is much more efficient than mesophilic bacteria or algae, and produced soluble monomer feedstocks [134].

Industrial fermentation at elevated temperature also reduces the chances of contamination or bacteriophage infection, which is common with mesophilic hosts such as *E. coli* or *Salmonella* [16]. Nevertheless, recombinant thermophilic enzymes can be expressed in a mesophile that is easier to grow in a fermenter and which has several technical advantages [44]: The recombinant enzymes can be easily purified by using a heat-treatment step, whereby most of the host bacterial proteins will denature and precipitate out; oftentimes, a thermophilic enzyme is also tolerant to chemical denaturants, such as guanidinium hydrochloride, which is useful if the recombinant needs to be extracted from inclusion bodies.

### 4.3. Biological Changes in Global Warming

#### 4.3.1. Individual Species

Natural selection, by definition, engender a fitter variant, which is, therefore, different from the original, and this would also apply to the survivors of global warming. The effect of warming on a variety of organisms across phylogeny has been documented; however, the underlying genetic and molecular determinants have remain largely unraveled, clearly due to the multiplicity and complexity of the genes involved. Most organisms that have survived so far have used mitigation strategies, generally changing their habitats and habits such as foraging places and periods [1,2,137]. It is common knowledge, for example, that most animals rest in the shades at mid-noon on the hot summer days, and forage or hunt in cooler periods. Unfortunately, despite its obvious life-saving value, mitigation without acquiring mutational resistance to heat is only a temporary solution that shifts the problem to another day for eventual extinction. Nonetheless, some of these phenotypic changes in thermal adaptation may be accompanied by genetic alterations that have not been fully delineated or interrogated for their long-term value in anthropogenic climate change [138]. A few examples, in which there is some gleaning of the molecular mechanism, are presented below.

Insects exhibit a complex phenotype of heat tolerance [139]. Perhaps counter-intuitively, insects often shrink in size and other morphological measurements as temperatures rises. Studies in the thermophilic ant [140], *Aphaenogaster senilis*, showed lack of larval development at the suboptimal temperature of 20 °C. At higher temperatures, developmental speed increased, but adults were smaller. At the same time, thermal resistance tests confirmed that ants raised at 28 and 32 °C had higher half-lethal temperatures than those raised at 24 °C. These results and others [141] show that the ants can use phenotypic plasticity to adjust to heat before genetic mutational selection occurs, and it is independent of morphological dimensions. This trait is obviously useful for the survival of the insect in rapid climate warming, but it currently lacks a mechanistic explanation. It would be interesting to test how the molecular features of *Aphaenogaster senilis* proteins compare with their homologs in other insects of lower or higher optimal growth temperatures.

Much more is known about chemical changes in the insect exoskeleton induced by heat. In a very recent study [142], three model but diverse ant species, namely *Atta sexdens, Ectatomma brunneum,* and *Odontomachus bauri,* were subjected to different temperatures for 5 h, and their fatty acids and cuticle hydrocarbon (CHC) contents were analyzed. None of the three was affected by temperatures below 30 °C, but each was differentially sensitive to higher temperatures. The capacity to survive was unrelated to fatty acids, but the CHCs showed significant quantitative and qualitative variation with temperature, having longer chain length of linear alkanes above 30 °C. These results are in agreement with the role of the cuticular exoskeleton of insects as a desiccation barrier, which is critical in preventing water loss at higher temperatures. The biosynthetic pathway of CHCs from fatty acids involves a complex network of “very long chain” fatty acid synthases, elongases, and desaturases, reductases, and finally, oxidative decarbonylases [143]. How the expression or activity of these enzymes is regulated by short-term heat exposure can be a highly rewarding area of research. It has been suggested that in the fatty acid synthase complex, the length of the fatty acid chain (usually 16-carbon-long palmitic acid) is sensed by a “molecular caliper” mechanism, in which the size of the hydrophobic pocket, where the synthase sits, determines the length, and that in the case of very long chain synthase, this pocket is larger [144]. With this model in mind, it is tempting to speculate that increased temperature may further enlarge this pocket, allowing a longer alkene chain to be formed. It is also worth testing whether the heat shock proteins play an as yet unknown role in this regulation, perhaps stabilizing the enlarged synthase.

At odds with our expectations, there are a few cases where a specific organism has actually benefited from global warming, at least in an isolated, short-term consideration. Higher underwater temperatures as a rule increase a plant’s demand for carbon, but at the same time, the accompanying floodwaters increase water turbidity and lower the light levels, compromising photosynthesis, and hence, creating carbon deficiency. The presence of multiple other stress factors accompanying a temperature shift creates a synergistic, lethal effect; however, for some seagrasses, warmer waters appear beneficial, resulting in faster reproduction, the basis of which remains to be studied [1]. Due to the complexity of multifactorial phenotypic changes and strain variations, it has been cautioned that climatic results without a mechanism may not have the predictive value expected of them [145,146,147]. It has been conjectured that the most extreme organisms may be found in exoplanets and atmospheres in outer space [148,149,150]. If indeed found, these alien organisms will most certainly reveal new molecular and metabolic features, but their very exotic nature will also make them nearly impossible to study in terrestrial laboratories.

It is important to realize that biological evolution in global warming is indeed a “global” phenomenon, in which selection of the fittest operates not only at the level of molecular, cellular, or individual species level, but also at the population level, which includes all types reproduction and genetic recombination, and perhaps also interaction between heterologous populations (e.g., “host–pathogen interactions”, Section 4.3.2). Thus, long-term survival is dictated by a concerted and complex interplay between genomic changes, animal behavior, and mitigation by migration. Nevertheless, as migration is the most immediate response of animals to temperature change, it has received significant attention. Habitat range shifts and range contractions have played particularly major roles in the history of all species; however, the molecular consequences of these processes have been investigated only recently. One population study [151] employed computer simulations to study the patterns of molecular diversity both within and between populations, and for multiple types of range contractions and shifts. The results revealed that changes in range contractions affect genetic diversity but the speed of change have contrasting effects; specifically, fast range contractions lead to higher diversity and lower genetic differentiation than slow contractions. This contrasts with the effect of range shifts: Fast shifts lead to lower levels of diversity, when compared to slow shifts. Overall, the results suggested that shifts and contractions of range interact in a complex but tractable manner and that the levels of diversity preserved after a climate change both within and between refuge areas will not only depend on the dispersal abilities of a species but also on the speed of the change. It also implies that, in any climatic change, species with different generation times will be impacted differently.

#### 4.3.2. Host–Pathogen Interactions

For many pathogens that are transmitted by vectors, such as tick-borne parasites, a visible change is their gradual migration to higher altitudes as the normal habitat of their vector gets warmer, which is again a mitigating response [12]; as mentioned before, a more universal adaptation to temperature can occur only by genetic changes in the host or the pathogen. The obligatory parasites, such as viruses, must evolve in co-ordination with the host cellular machinery, which they co-opt. The interaction of their mutually evolving protein domains is currently an interesting area of structural and biochemical analysis, as shown recently [152]. Infectious pathogens that are also free-living, such as the disease-causing bacteria, yeast and fungi, and some unicellular protozoa (e.g., Trypanosomes), can evolve to encode heat-tolerant mutations independent of their hosts. However, for optimal infection, they too need to fit to the pathways of the thermotolerant host, in which the mutations to heat-resistance may have produced subtle changes in their interaction with the cognate parasite function.

Important molecular mechanisms in this area have been gleaned from viral pathogens, in part because host–virus interactions lend themselves to tractable genetic and biochemical assays. Viruses are by far the most abundant pathogens in the world’s oceans that are estimated to contain 10^30^ virions that infect diverse marine organisms [153]. The large pool not only allows for host–virus co-evolution, but also for the generation of novel virus strains by genetic assortment, such as the recent discovery of apparently novel coronaviruses in beluga whale and dolphins [154,155,156]. A case of apparent benefit of the host has been noted in *Emiliania huxleyi*, a ubiquitous marine phytoplankton, responsible for dense annual blooms that have a profound impact on planetary carbon and sulfur cycles [157]. The blooms are regularly terminated by dsDNA viruses known as *E. huxleyi* Viruses or EhVs. In an interesting recent study, *E. huxleyi* was found to develop EhV-resistance when the temperature increased by only 3 °C; this also resulted in increased production of dimethyl sulfide, a major plankton metabolite that forms “cloud condensation nuclei”, a global rain-maker. The authors proposed that alterations to the *E. huxleyi* surface receptors are responsible for the temperature-induced resistance.

Finally, there are many other peripheral areas of global warming that will also affect biological life, such as possible alterations of light intensity and circadian rhythm, and change of the environment from aerobic to anaerobic [158,159,160,161], which will add to the burden of selection.

## 5. Summary

**Figure 5:**
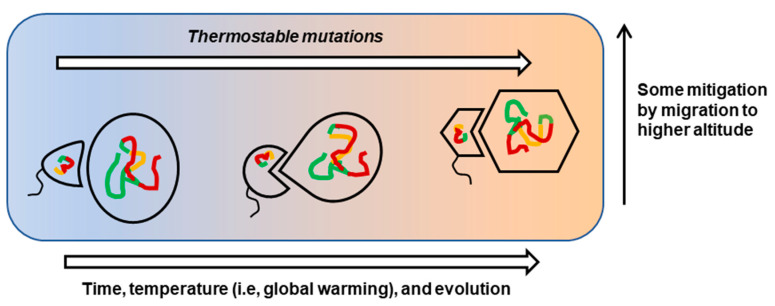
Selection for thermostable mutations, as well as mitigation by organismal behavior and habitat (range) change, in global warming. A pathogen (the smaller structure with flagella) and its host (the larger structure) are shown as a pair, to indicate that they will mutate together, with increasing temperature, as time passes. The changes are indicated by the altered structure of the protein (shown in three colors for the domains, to match Figure 3) and possibly by altered morphology. Simultaneously, the organisms may migrate to higher habitats, for a cooler atmosphere.

It should be noted that in spite of the most careful and methodical analyses, the complex nature of the protein folding ensemble, entropy landscape, and kinetic parameters makes it nearly impossible to derive an invariable universal set of rules for thermotolerance in all organisms or all proteins. As we have seen in the review, practically all molecular parameters derived from thermotolerance studies have some exceptions [70]. Nonetheless, these “nearly universal” molecular signature(s) will form a useful framework against which essentially all evolutionary selections in climate change can be interrogated. A simplified summary of interactive molecular changes is schematically depicted here (Figure 5).

As indicated in the diagram, mutation and mitigation will both work concurrently, the first as a genetic change and the second as an immediate behavioral reaction. In persistent warming, various forms of life will prevail, but radical mutational changes will create highly modified, newer organisms, many of which will be morphologically different, with altered community structures [162]. Invasive and predatory species may also take over as privileged survivors. Finally, in terms of novel mechanisms, it is tempting to postulate that molecular changes may also result from the non-genetic mechanisms (i.e., without genomic mutations) that include but are not limited to the following: (a) transcriptional or translation induction of a protein by a thermo-sensing mechanism, not unlike heat-shock response; (b) processing of a pro-enzyme into its active form; (c) alternative splicing, promoted by a heat-induced splicing factor; (d) altered RNA fold; (e) post-translational modifications, such as phosphorylation or methylation; (f) change of conformation of a protein, accompanying temperature changes [163], for which dramatic examples are known, e.g., lymphotactin [164]; and (g) epigenetic changes in the genome. These will constitute novel areas of future investigation of molecular biological strategies adopted by living beings in their effort to survive global warming.

## References

[1] Root T.L., Price J.T., Hall K.R., Schneider S.H., Rosenzweig C., Pounds J.A. (2003). Fingerprints of global warming on wild animals and plants. Nature.

[2] Klausmeyer K.R., Shaw M.R. (2009). Climate change, habitat loss, protected areas and the climate adaptation potential of species in Mediterranean ecosystems worldwide. PLoS ONE.

[3] Hughes L. (2000). Biological consequences of global warming: Is the signal already apparent?. Trends Ecol. Evol..

[4] Benton M.J. (2018). Hyperthermal-driven mass extinctions: Killing models during the Permian–Triassic mass extinction. Philos. Trans. A Math. Phys. Eng. Sci..

[5] Madeira D., Araújo J.E., Vitorino R., Capelo J.L., Vinagre C., Diniz M.S. (2016). Ocean warming alters cellular metabolism and induces mortality in fish early life stages: A proteomic approach. Environ. Res..

[6] Traboni C., Mammola S.D., Ruocco M., Ontoria Y., Ruiz J.M., Procaccini G., Marín-Guirao L. (2018). Investigating cellular stress response to heat stress in the seagrass *Posidonia oceanica* in a global change scenario. Mar. Environ. Res..

[7] Faldyn M.J., Hunter M.D., Elderd B.D. (2018). Climate change and an invasive, tropical milkweed: An ecological trap for monarch butterflies. Ecology.

[8] Bjorkman A.D., Myers-Smith I.H., Elmendorf S.C., Normand S., Rüger N., Beck P.S., Blach-Overgaard A., Blok D., Cornelissen J.H.C., Forbes B.C. (2018). Plant functional trait change across a warming tundra biome. Nature.

[9] Nolan C., Overpeck J.T., Allen J.R., Anderson P.M., Betancourt J.L., Binney H.A., Brewer S., Bush M.B., Chase B.M., Cheddadi R. (2018). Past and future global transformation of terrestrial ecosystems under climate change. Science.

[10] Grubb M. (2003). The economics of the Kyoto protocol. World Econ..

[11] Spash C.L. (2010). The brave new world of carbon trading. New Political Econ..

[12] Rossati A. (2017). Global warming and its health impact. Int. J. Occup. Environ. Med..

[13] Stetter K.O. (2006). History of discovery of the first hyperthermophiles. Extremophiles.

[14] Goncearenco A., Ma B.G., Berezovsky I.N. (2014). Molecular mechanisms of adaptation emerging from the physics and evolution of nucleic acids and proteins. Nucleic Acids Res..

[15] Brininger C., Spradlin S., Cobani L., Evilia C. (2018). The more adaptive to change, the more likely you are to survive: Protein adaptation in extremophiles. Semin. Cell. Dev. Biol..

[16] Zeldes B.M., Keller M.W., Loder A.J., Straub C.T., Adams M.W.W., Kelly R.M. (2015). Extremely thermophilic microorganisms as metabolic engineering platforms for production of fuels and industrial chemicals. Front. Microbiol..

[17] Turner P., Mamo G., Karlsson E.N. (2007). Potential and utilization of thermophiles and thermostable enzymes in biorefining. Microb. Cell Fact..

[18] Maheshwari R., Bharadwaj G., Bhat M.K. (2000). Thermophilic fungi: Their physiology and enzymes. Microbiol. Mol. Biol. Rev..

[19] Kim H., Kim S., Jung Y., Han J., Yun J.-H., Chang I., Lee W. (2016). Probing the folding-unfolding transition of a thermophilic protein, MTH1880. PLoS ONE.

[20] Daggett V., Fersht A.R. (2003). Is there a unifying mechanism for protein folding?. Trends Biochem. Sci..

[21] Fersht A.R., Sato S. (2004). Phi-value analysis and the nature of protein-folding transition states. Proc. Natl. Acad. Sci. USA.

[22] Gianni S., Ivarsson Y., Jemth P., Brunori M., Travaglini-Allocatelli C. (2007). Identification and characterization of protein folding intermediates. Biophys. Chem..

[23] Adams B., Musiyenko A., Kumar R., Barik S. (2005). A novel class of dual-family immunophilins. J. Biol. Chem..

[24] Joshi N., Raveendran A., Nagotu S. (2020). Chaperones and proteostasis: Role in Parkinson’s Disease. Diseases.

[25] Mallik S., Kundu S. (2013). A comparison of structural and evolutionary attributes of *Escherichia coli* and *Thermus thermophilus* small ribosomal subunits: Signatures of thermal adaptation. PLoS ONE.

[26] Polley S., Jana B., Chakrabarti G., Sau S. (2014). Inhibitor-induced conformational stabilization and structural alteration of a mip-like peptidyl prolyl cis-trans isomerase and its C-terminal domain. PLoS ONE.

[27] Isticato R., Lanzilli M., Petrillo C., Donadio G., Baccigalupi L., Ricca E. (2020). *Bacillus subtilis* builds structurally and functionally different spores in response to the temperature of growth. Environ. Microbiol..

[28] Vieille C., Burdette D.S., Zeikus J.G. (1996). Thermozymes. Biotechnol. Annu. Rev..

[29] Grättinger M., Dankesreiter A., Schurig H., Jaenicke R. (1998). Recombinant phosphoglycerate kinase from the hyperthermophilic bacterium *Thermotoga maritima*: Catalytic, spectral and thermodynamic properties. J. Mol. Biol..

[30] Bauer M.W., Kelly R.M. (1998). The family 1b-glucosidases from *Pyrococcus furiosus* and *Agrobacterium faecalis* share a common catalytic mechanism. Biochemistry.

[31] Vieille C., Hess J.M., Kelly R.M., Zeikus J.G. (1995). xylA cloning and sequencing and biochemical characterization of xylose from *Thermotoga neapolitana*. Appl. Environ. Microbiol..

[32] Zwickl P., Fabry S., Bogedain C., Hass A., Hensel R. (1990). Glyceraldehyde-3-phosphate dehydrogenase from the hyperthermophilic archaebacterium *Pyrococcus woesei*: Characterization of the enzyme, cloning and sequencing of the gene, and expression in *Escherichia coli*. J. Bacteriol..

[33] Auerbach G., Ostendorp R., Prade L., Korndörfer L., Dams T., Huber R., Jaenicke R. (1998). Lactate dehydrogenase from the hyperthermophilic bacterium *Thermotoga maritima*: The crystal structure at 2.1 A resolution reveals strategies for intrinsic protein stabilization. Structure.

[34] Chi Y.I., Martinez-Cruz L.A., Jancarik J., Swanson R.V., Robertson D.E., Kim S.H. (1999). Crystal structure of the beta-glycosidase from the hyperthermophile *Thermosphaera aggregans*: Insights into its activity and thermostability. FEBS Lett..

[35] Tahirov T.H., Oki H., Tsukihara T., Ogasahara K., Yutani K., Ogata K., Izu Y., Tsunasawa S., Kato I. (1998). Crystal structure of methionine aminopeptidase from hyperthermophile, *Pyrococcus furiosus*. J. Mol. Biol..

[36] Russell R.J., Ferguson J.M., Hough D.W., Danson M.J., Taylor G.L. (1997). The crystal structure of citrate synthase from the hyperthermophilic archaeon *Pyrococcus furiosus* at 1.9A resolution. Biochemistry.

[37] Morimoto R.I. (1993). Cells in stress: Transcriptional activation of heat shock genes. Science.

[38] Richter K., Haslbeck M., Buchner J. (2010). The heat shock response: Life on the verge of death. Mol. Cell.

[39] Kumsta C., Chang J.T., Schmalz J., Hansen M. (2017). Hormetic heat stress and HSF-1 induce autophagy to improve survival and proteostasis in *C. elegans*. Nat. Commun..

[40] Gould S.J., Eldredge N. (1993). Punctuated equilibrium comes of age. Nature.

[41] Bloom J.D., Labthavikul S.T., Otey C.R., Arnold F.H. (2006). Protein stability promotes evolvability. Proc. Natl. Acad. Sci. USA.

[42] Shoichet B.K., Baase W.A., Kuroki R., Matthews B.W. (1995). A relationship between protein stability and protein function. Proc. Natl. Acad. Sci. USA.

[43] Tokuriki N., Stricher F., Serrano L., Tawfik D.S. (2008). How protein stability and new functions trade off. PLoS Comp. Biol..

[44] Slatkina M., Racimoa F. (2016). Ancient DNA and human history. Proc. Natl. Acad. Sci. USA.

[45] Harms M.J., Thornton J.W. (2010). Analyzing protein structure and function using ancestral gene reconstruction. Curr. Opin. Struct. Biol..

[46] Arenas M., Sánchez-Cobos A., Bastolla U. (2015). Maximum-likelihood phylogenetic inference with selection on protein folding stability. Mol. Biol. Evol..

[47] Akanuma S. (2017). Characterization of reconstructed ancestral proteins suggests a change in temperature of the ancient biosphere. Life.

[48] Arenas M., Weber C.C., Liberles D.A., Bastolla U. (2017). ProtASR: An evolutionary framework for ancestral protein reconstruction with selection on folding stability. Syst. Biol..

[49] Garcia A.K., Kaçar B. (2019). How to resurrect ancestral proteins as proxies for ancient biogeochemistry. Free Radic. Biol. Med..

[50] Akanuma S., Nakajima Y., Yokobori S.-I., Kimura M., Nemoto N., Mase T., Miyazono K.-I., Tanokura M., Yamagishi A. (2013). Experimental evidence for the thermophilicity of ancestral life. Proc. Natl. Acad. Sci. USA.

[51] Weiss M.C., Sousa F.L., Mrnjavac N., Neukirchen S., Roettger M., Nelson-Sathi S., Martin W.F. (2016). The physiology and habitat of the last universal common ancestor. Nat. Microbiol..

[52] Nguyen V., Wilson C., Hoemberger M., Stiller J.B., Agafonov R.V., Kutter S., English J., Theobald D.L., Kern D. (2017). Evolutionary drivers of thermoadaptation in enzyme catalysis. Science.

[53] Fralick P., Carter J.E. (2011). Neoarchean deep marine paleotemperature: Evidence from turbidite successions. Precambrian Res..

[54] Achenbach-Richter L., Gupta R., Zillig W., Woese C.R. (1988). Rooting the archaebacterial tree: The pivotal role of *Thermococcus celer* in archaebacterial evolution. Syst. Appl. Microbiol..

[55] Ciccarelli F.D., Doerks T., von Mering C., Creevey C.J., Snel B., Bork P. (2006). Toward automatic reconstruction of a highly resolved tree of life. Science.

[56] Barik S. (2017). On the role, ecology, phylogeny, and structure of dual-family immunophilins. Cell Stress Chaperones.

[57] Semba Y., Ishida M., Yokobori S.-I., Yamagishi A. (2015). Ancestral amino acid substitution improves the thermal stability of recombinant lignin-peroxidase from white-rot fungi, *Phanerochaete chrysosporium* strain UAMH 3641. Protein Eng. Des. Sel..

[58] Iwabata H., Watanabe K., Ohkuri T., Yokobori S.-I., Yamagishi A. (2005). Thermostability of ancestral mutants of *Caldococcus noboribetus* isocitrate dehydrogenase. FEMS Microbiol. Lett..

[59] Miyazaki J., Nakaya S., Suzuki T., Tamakoshi M., Oshima T., Yamagishi A. (2001). Ancestral residues stabilizing 3-isopropylmalate dehydrogenase of an extreme thermophile: Experimental evidence supporting the thermophilic common ancestor hypothesis. J. Biochem..

[60] Gaucher E.A., Govindarajan S., Ganesh O.K. (2008). Palaeotemperature trend for Precambrian life inferred from resurrected proteins. Nature.

[61] Perez-Jimenez R., Inglés-Prieto A., Zhao Z.M., Sanchez-Romero I., Alegre-Cebollada J., Kosuri P., Garcia-Manyes S., Kappock T.J., Tanokura M., Holmgren A. (2011). Single-molecule paleoenzymology probes the chemistry of resurrected enzymes. Nat. Struct. Mol. Biol..

[62] Fukuda Y., Abe A., Tamura T., Kishimoto T., Sogabe A., Akanuma S., Yokobori S.-I., Yamagishi A., Imada K., Inagaki K. (2016). Epistasis effects of multiple ancestral-consensus amino acid substitutions on the thermal stability of glycerol kinase from Cellulomonas sp. NT3060. J. Biosci. Bioeng..

[63] Furukawa R., Toma W., Yamazaki K., Akanuma S. (2020). Ancestral sequence reconstruction produces thermally stable enzymes with mesophilic enzyme-like catalytic properties. Sci. Rep..

[64] Vieille C., Zeikus G.J. (2001). Hyperthermophilic enzymes: Sources, uses, and molecular mechanisms for thermostability. Microbiol. Mol. Biol. Rev..

[65] Watters A.L., Baker D. (2004). Searching for folded proteins in vitro and in silico. Eur. J. Biochem..

[66] Lesk A.M., Chothia C. (1980). Solvent accessibility, protein surfaces, and protein folding. Biophys. J..

[67] Chothia C., Levitt M., Richardson D. (1981). Helix to helix packing in proteins. J. Mol. Biol..

[68] Gerstein M., Lesk A.M., Chothia C. (1994). Structural mechanisms for domain movements in proteins. Biochemistry.

[69] Baker D. (2019). What has *de novo* protein design taught us about protein folding and biophysics?. Protein Sci..

[70] Hait S., Mallik S., Basu S., Kundu S. (2020). Finding the generalized molecular principles of protein thermal stability. Proteins.

[71] Zeldovich K.B., Berezovsky I.N., Shakhnovich E.I. (2007). Protein and DNA sequence determinants of thermophilic adaptation. PLoS Comput. Biol..

[72] Miralles F. (2011). Compositional and structural features related to thermal stability in the archaea SRP19 and SRP54 signal recognition particle proteins. J. Mol. Evol..

[73] Pack S.P., Yoo Y.J. (2004). Protein thermostability: Structure-based difference of amino acid between thermophilic and mesophilic proteins. J. Biotechnol..

[74] Kumar S., Tsai C.-J., Nussinov R. (2000). Factors enhancing protein thermostability. Protein Eng. Des. Sel..

[75] Mizuguchi K., Sele M., Cubellis M.V. (2007). Environment specific substitution tables for thermophilic proteins. BMC Bioinform..

[76] Khan M.F., Patra S. (2018). Deciphering the rationale behind specific codon usage pattern in extremophiles. Sci. Rep..

[77] Petukhov M., Kil Y., Kuramitsu S., Lanzov V. (1997). Insights into thermal resistance of proteins from the intrinsic stability of their α-helices. Proteins.

[78] Szilágyi A., Závodszky P. (2000). Structural differences between mesophilic, moderately thermophilic and extremely thermophilic protein subunits: Results of a comprehensive survey. Structure.

[79] Bowie J.U. (2005). Solving the membrane protein folding problem. Nature.

[80] Minetti C.A.S., Remeta D.P. (2006). Energetics of membrane protein folding and stability. Arch. Biochem. Biophys..

[81] Matthews B.W., Nicholson H., Becktel W.J. (1987). Enhanced protein thermostability from site-directed mutations that decrease the entropy of unfolding. Proc. Natl. Acad. Sci. USA.

[82] Sadeghi M., Naderi-Manesh H., Zarrabi M., Ranjbar B. (2006). Effective factors in thermostability of thermophilic proteins. Biophys. Chem..

[83] Zhou X.-X., Wang Y.-B., Pan Y.-J., Li W.-F. (2008). Differences in amino acids composition and coupling patterns between mesophilic and thermophilic proteins. Amino Acids.

[84] Greaves R.B., Warwicker J. (2009). Stability and solubility of proteins from extremophiles. Biochem. Biophys. Res. Commun..

[85] Haney P.J., Badger J.H., Buldak G.L., Reich C.I., Woese C.R., Olsen G.J. (1999). Thermal adaptation analyzed by comparison of protein sequences from mesophilic and extremely thermophilic *Methanococcus* species. Proc. Natl. Acad. Sci. USA.

[86] Chao Y.-C., Merritt M., Schaefferkoetter D., Evans T.G. (2020). High-throughput quantification of protein structural change reveals potential mechanisms of temperature adaptation in *Mytilus* mussels. BMC Evol. Biol..

[87] Taylor T.J., Vaisman I.I. (2010). Discrimination of thermophilic and mesophilic proteins. BMC Struct. Biol..

[88] Sterpone F., Bertonati C., Briganti G., Melchionna S. (2009). Key role of proximal water in regulating thermostable proteins. J. Phys. Chem. B.

[89] Mou Z., Ding Y., Wang X., Cai Y. (2014). Comparison of protein-water interactions in psychrophilic, mesophilic, and thermophilic Fe-SOD. Protein Pept. Lett..

[90] Saunders N.F.W., Thomas T., Curmi P.M.G., Mattick J.S., Kuczek E., Slade R., Davis J., Franzmann P.D., Boone D., Rusterholtz K. (2003). Mechanisms of thermal adaptation revealed from the genomes of the Antarctic archaea *Methanogenium frigidum* and *Methanococcoides burtonii*. Genome Res..

[91] Karshikoff A., Ladenstein R. (2001). Ion pairs and the thermotolerance of proteins from hyperthermophiles: A “Traffic Rule” for hot roads. Trends Biochem. Sci..

[92] Robinson-Rechavi M., Alibés A., Godzik A. (2006). Contribution of electrostatic interactions, compactness and quaternary structure to protein thermostability: Lessons from structural genomics of *Thermotoga maritima*. J. Mol. Biol..

[93] Panja A.S., Maiti S., Bandyopadhyay B. (2020). Protein stability governed by its structural plasticity is inferred by physicochemical factors and salt bridges. Sci. Rep..

[94] Sternke M., Tripp K.W., Barrick D. (2019). Consensus sequence design as a general strategy to create hyperstable, biologically active proteins. Proc. Natl. Acad. Sci. USA.

[95] Okada J., Okamoto T., Mukaiyama A., Tadokoro T., You D.-J., Chon H., Koga Y., Takano K., Kanaya S. (2010). Evolution and thermodynamics of the slow unfolding of hyperstable monomeric proteins. BMC Evol. Biol..

[96] Gromiha M.M., Pathak M.C.D., Saraboji K., Ortlund E.A., Gaucher E.A. (2013). Hydrophobic environment is a key factor for the stability of thermophilic proteins. Proteins.

[97] Takano K., Aoi A., Koga Y., Kanaya S. (2013). Evolvability of thermophilic proteins from archaea and bacteria. Biochemistry.

[98] Pace A.L., Wong R.L., Zhang Y.T., Kao Y.H., Wang Y.J. (2013). Asparagine deamidation dependence on buffer type, pH, and temperature. J. Pharm. Sci..

[99] Saelensminde G., Halskau Ø., Jonassen I. (2009). Amino acid contacts in proteins adapted to different temperatures: Hydrophobic interactions and surface charges play a key role. Extremophiles.

[100] Barik S. (2020). The uniqueness of tryptophan in biology: Properties, metabolism, interactions and localization in proteins. Int. J. Mol. Sci..

[101] Main E.R.G., Xiong Y., Cocco M.J., D’Andrea L., Regan L. (2003). Design of stable alpha-helical arrays from an idealized TPR motif. Structure.

[102] Barik S. (2019). Protein tetratricopeptide repeat and the companion non-tetratricopeptide repeat helices: Bioinformatic analysis of interhelical interactions. Bioinform. Biol. Insights.

[103] Barik S. (2020). The nature and arrangement of pentatricopeptide domains and the linker sequences between them. Bioinform. Biol. Insights.

[104] Kajava A.V. (2012). Tandem repeats in proteins: From sequence to structure. J. Struct. Biol..

[105] Barkan A., Small I. (2014). Pentatricopeptide repeat proteins in plants. Annu. Rev. Plant. Biol..

[106] Perez-Riba A., Itzhaki L.S. (2019). The tetratricopeptide-repeat motif is a versatile platform that enables diverse modes of molecular recognition. Curr. Opin. Struct. Biol..

[107] Sawyer N., Chen J., Regan L. (2013). All repeats are not equal: A module-based approach to guide repeat protein design. J. Mol. Biol..

[108] Tomazic S.J., Klibanov A.M. (1988). Mechanisms of irreversible thermal inactivation of Bacillus alpha-amylases. J. Biol. Chem..

[109] Flaugh S.L., Mills I.A., King J. (2006). Glutamine deamidation destabilizes human gammaD-crystallin and lowers the kinetic barrier to unfolding. J. Biol. Chem..

[110] Soulby A.J., Heal J.W., Barrow M.P., Roemer R.A., O’Connor P.B. (2015). Does deamidation cause protein unfolding? A top-down tandem mass spectrometry study. Protein Sci..

[111] Chen H.M., Ford C., Reilly P.J. (1994). Substitution of asparagine residues in *Aspergillus awamori* glucoamylase by site-directed mutagenesis to eliminate N-glycosylation and inactivation by deamidation. Biochem. J..

[112] Sriprapundh D., Vieille C., Zeikus J.G. (2000). Molecular determinants of xylose isomerase thermal stability and activity: Analysis of thermozymes by site-directed mutagenesis. Protein Eng..

[113] Zhang Y., Porcelli M., Cacciapuoti G., Ealick S.E. (2006). The crystal structure of 5′-deoxy-5′-methylthioadenosine phosphorylase II from *Sulfolobus solfataricus*, a thermophilic enzyme stabilized by intramolecular disulfide bonds. J. Mol. Biol..

[114] Wang C.-H., Hsu C.-H., Wu Y.-M., Luo Y.-C., Tu M.-H., Chang W., Cheng R.H., Lin C.-S. (2010). Roles of cysteines Cys115 and Cys201 in the assembly and thermostability of grouper betanodavirus particles. Virus Genes.

[115] Pecher P., Arnold U. (2009). The effect of additional disulfide bonds on the stability and folding of ribonuclease A. Biophys. Chem..

[116] Liu L., Deng Z., Yang H., Li J., Shin H.-d., Chen R.R., Du G., Chen J. (2014). In silico rational design and systems engineering of disulfide bridges in the catalytic domain of an alkaline α-amylase from *Alkalimonas amylolytica* to improve thermostability. Appl. Environ. Microbiol..

[117] Yang J., Yan R., Roy A., Xu D., Poisson J., Zhang Y. (2015). The I-TASSER suite: Protein structure and function prediction. Nat. Methods.

[118] Finch A.J., Kim J.R. (2018). Thermophilic proteins as versatile scaffolds for protein engineering. Microorganisms.

[119] Petsko G.A. (2001). Structural basis of thermostability in hyperthermophilic proteins, or “there’s more than one way to skin a cat”. Methods Enzymol..

[120] Camps M., Herman A., Loh E., Loeb L.A. (2007). Genetic constraints on protein evolution. Crit. Rev. Biochem. Mol. Biol..

[121] Razvi A., Scholtz J.M. (2006). Lessons in stability from thermophilic proteins. Protein Sci..

[122] DePristo M.A., Weinreich D.M., Hartl D.L. (2005). Missense meanderings in sequence space: A biophysical view of protein evolution. Nat. Rev. Genet..

[123] Glyakina A.V., Galzitskaya O.V. (2020). How quickly do proteins fold and unfold, and what structural parameters correlate with these values?. Biomolecules.

[124] Arnold F.H. (2009). How proteins adapt: Lessons from directed evolution. Cold Spring Harb. Symp. Quant. Biol..

[125] Karshikoff A., Nilsson L., Ladenstein R. (2015). Rigidity versus flexibility: The dilemma of understanding protein thermal stability. FEBS J..

[126] Riehle M.M., Bennett A.F., Long A.D. (2001). Genetic architecture of thermal adaptation in *Escherichia coli*. Proc. Natl. Acad. Sci. USA.

[127] Shi B., Xia X. (2005). Genetic variation in clones of *Pseudomonas pseudoalcaligenes* after ten months of selection in different thermal environments in the laboratory. Curr. Microbiol..

[128] Tamakoshi M., Yamagishi A., Oshima T. (1995). Screening of stable proteins in an extreme thermophile, *Thermus thermophilus*. Mol. Microbiol..

[129] Perl D., Mueller U., Heinemann U., Schmid F.X. (2000). Two exposed amino acid residues confer thermostability on a cold shock protein. Nat. Struct. Biol..

[130] Akanuma S., Yamagishi A., Tanaka N., Oshima T. (1998). Serial increase in the thermal stability of 3-isopropylmalate dehydrogenase from *Bacillus subtilis* by experimental evolution. Protein Sci..

[131] Blaby I.K., Lyons B.J., Wroclawska-Hughes E., Phillips G.C.F., Pyle T.P., Chamberlin S.G., Benner S.A., Lyons T.J., de Crécy-Lagard V., de Crécy E. (2012). Experimental evolution of a facultative thermophile from a mesophilic ancestor. Appl. Environ. Microbiol..

[132] Estes J.A., Terborgh J., Brashares J.S., Power M.E., Berger J., Bond W.J., Carpenter S.R., Essington T.E., Holt R.D., Jackson J.B.C. (2011). Trophic downgrading of planet Earth. Science.

[133] Seo H., Lee J.-W., Garcia S., Trinh C.T. (2019). Single mutation at a highly conserved region of chloramphenicol acetyltransferase enables isobutyl acetate production directly from cellulose by *Clostridium thermocellum* at elevated temperatures. Biotechnol. Biofuels.

[134] Yan F., Wei R., Cui Q., Bornscheuer U.T., Liu Y.-J. (2020). Thermophilic whole-cell degradation of polyethylene terephthalate using engineered *Clostridium thermocellum*. Microb. Biotechnol..

[135] Liao Y., Williams T.J., Walsh J.C., Ji M., Poljak A., Curmi P.M.G., Duggin I.G., Cavicchioli R. (2016). Developing a genetic manipulation system for the Antarctic archaeon, *Halorubrum lacusprofundi*: Investigating acetamidase gene function. Sci. Rep..

[136] Deng H., Wei R., Luo W., Hu L., Li B., Di Y., Shi H. (2020). Microplastic pollution in water and sediment in a textile industrial area. Environ. Pollut..

[137] Moritz M., Behnke R., Beitl C.M., Bird R.B., Chiaravalloti R.M., Clark J.K., Crabtree S.A., Downey S.S., Hamilton I.M., Phang S.C. (2018). Emergent sustainability in open property regimes. Proc. Natl. Acad. Sci. USA.

[138] Rane R.V., Pearce S.L., Li F., Coppin C., Schiffer M., Shirriffs J., Sgrò C.M., Griffin P.C., Zhang G., Lee S.F. (2019). Genomic changes associated with adaptation to arid environments in cactophilic *Drosophila* species. BMC Genom..

[139] Baudier K., O’Donnell S. (2018). Complex body size differences in thermal tolerance among army ant workers (*Eciton burchellii parvispinum*). J. Therm. Biol..

[140] Oms C.S., Cerdá X., Boulay R. (2017). Is phenotypic plasticity a key mechanism for responding to thermal stress in ants?. Naturwissenschaften.

[141] Otte T., Hilker M., Geiselhardt S. (2018). Phenotypic plasticity of cuticular hydrocarbon profiles in insects. J. Chem. Ecol..

[142] Duarte B.F., Michelutti K.B., Antonialli-Junior W.F., Cardoso C.A.L. (2019). Effect of temperature on survival and cuticular composition of three different ant species. J. Therm. Biol..

[143] Qiu Y., Tittiger C., Wicker-Thomas C., Goff G.L., Young S., Wajnberg E., Fricaux T., Taquet N., Blomquist G.J., Feyereisen R. (2012). An insect-specific P450 oxidative decarbonylase for cuticular hydrocarbon biosynthesis. Proc. Natl. Acad. Sci. USA.

[144] Denic V., Weissman J.S. (2007). A molecular caliper mechanism for determining very long-chain fatty acid length. Cell.

[145] Helmuth B., Kingsolver J.G., Carrington E. (2005). Biophysics, physiological ecology, and climate change: Does mechanism matter?. Annu. Rev. Physiol..

[146] Calosi P., Bilton D.T., Spicer J.I. (2008). Thermal tolerance, acclimatory capacity and vulnerability to global climate change. Biol. Lett..

[147] Mazzucco R., Nolte V., Vijayan T., Schlötterer C. (2020). Long-term dynamics among *Wolbachia* strains during thermal adaptation of their *Drosophila melanogaster* hosts. Front. Genet..

[148] Pikuta E.V., Hoover R.B., Tang J. (2007). Microbial extremophiles at the limits of life. Crit. Rev. Microbiol..

[149] Schwieterman E.W., Kiang N.Y., Parenteau M.N., Harman C.E., DasSarma S., Fisher T.M., Arney G.N., Hartnett H.E., Reinhard C.T., Olson S.L. (2018). Exoplanet biosignatures: A review of remotely detectable signs of life. Astrobiology.

[150] Schulze-Makuch D., Airo A., Schirmack J. (2017). The adaptability of life on Earth and the diversity of planetary habitats. Front. Microbiol..

[151] Arenas M., Ray N., Currat M., Excoffier L. (2012). Consequences of range contractions and range shifts on molecular diversity. Mol. Biol. Evol..

[152] Hashimoto Y., Sheng X., Murray-Nerger L.A., Cristea I.M. (2020). Temporal dynamics of protein complex formation and dissociation during human cytomegalovirus infection. Nat. Commun..

[153] Suttle C.A. (2007). Marine viruses–major players in the global ecosystem. Nat. Rev. Microbiol..

[154] Mihindukulasuriya K.A., Wu G., St Leger J., Nordhausen R.W., Wang D. (2008). Identification of a novel coronavirus from a beluga whale by using a panviral microarray. J. Virol..

[155] Woo P.C.Y., Lau S.K., Lam C.S., Tsang A.K., Hui S.W., Fan R.Y., Martelli P., Yuen K.Y. (2014). Discovery of a novel bottlenose dolphin coronavirus reveals a distinct species of marine mammal coronavirus in Gammacoronavirus. J. Virol..

[156] Barik S. (2020). Genus-specific pattern of intrinsically disordered central regions in the nucleocapsid protein of coronaviruses. Comput. Struct. Biotechnol. J..

[157] Kendrick B.J., DiTullio G.R., Cyronak T.J., Fulton J.M., Van Mooy B.A.S., Bidle K.D. (2014). Temperature-induced viral resistance in *Emiliania huxleyi* (*Prymnesiophyceae*). PLoS ONE.

[158] Harvell C.D., Mitchell C.E., Ward J.R., Altizer S., Dobson A.P., Ostfeld R.S., Samuel M.D. (2002). Climate warming and disease risks for terrestrial and marine biota. Science.

[159] Piedade G.J., Wesdorp E.M., Montenegro-Borbolla E., Maat D.S., Brussaard C.P.D. (2018). Influence of irradiance and temperature on the virus MpoV-45T infecting the arctic picophytoplankter *Micromonas polaris*. Viruses.

[160] Barik S. (2019). Molecular interactions between pathogens and the circadian clock. Int. J. Mol. Sci..

[161] Urnowey S., Ansai T., Bitko V., Barik S. (2020). Regulation of NF-kappa B and cell death by bacterial gingipains. BioRxiv.

[162] Dziuba M.K., Herdegen-Radwan M., Pluta E., Wejnerowski Ł., Szczuciński W., Cerbin S. (2020). Temperature increase altered Daphnia community structure in artificially heated lakes: A potential scenario for a warmer future. Sci. Rep..

[163] Bryan P.N., Orban J. (2010). Proteins that switch folds. Curr. Opin. Struct. Biol..

[164] Tuinstra R.L., Peterson F.C., Kutlesa S., Elgin E.S., Kron M.A., Volkman B.F. (2008). Interconversion between two unrelated protein folds in the lymphotactin native state. Proc. Natl. Acad. Sci. USA.

